# Novel Optical Modulator Photonic Device Based on TiN/Ti_3_C_2_ Heterojunction

**DOI:** 10.3390/s24165190

**Published:** 2024-08-11

**Authors:** Zexin Zhou, Miao Yan, Hu Liang, Jie Yu, Qidong Liu, Yufeng Song, Jianhua Ji, Zhenhong Wang, Ke Wang

**Affiliations:** 1 College of Electronics and Information Engineering, Shenzhen University, Shenzhen 518060, China; 2310434034@email.szu.edu.cn (Z.Z.); 2110436146@email.szu.edu.cn (Q.L.); yfsong@szu.edu.cn (Y.S.); jjh@szu.edu.cn (J.J.); wangzhenhong@szu.edu.cn (Z.W.); 2 Tianjin Navigation Instruments Research Institute, Tianjin 300131, China; pipo1999@sina.com (M.Y.); henda1214@163.com (J.Y.)

**Keywords:** TiN/Ti_3_C_2_ heterojunction, thermo-optic effect, all-optical modulators, Michelson interference

## Abstract

Due to the ability of optical modulators to achieve rapid modulation of optical signals, meeting the demands of high-speed data transmission, modulators based on different novel nanomaterials have become one of the research hotspots over the past dacade. Recently, TiN/Ti_3_C_2_ heterojunction exhibits highly efficient thermo-optic performance and extremely strong stability. Therefore, we have demonstrated an all-optical modulator based on the principle of Michelson interference and the thermo-optic effect in this paper. The modulator employs a TiN/Ti_3_C_2_ heterojunction-coated microfiber (THM) and further demonstrates its ability to generate phase shifts through an ASE light source. The modulator, with a phase shift slope of 0.025π/mW, can also convert the phase shifts of signal light into amplitude modulation through Michelson interference. The fixed signal light wavelength is 1552.09 nm, and the modulation depth is stable at about 26.4 dB within a wavelength detuning range of −10 to 6 nm; The waveforms of signal light at modulation rates of 500 Hz, 1000 Hz, 2000 Hz, and 3000 Hz were tested, and a 3 dB modulation bandwidth of 2 kHz was measured. The all-optical modulator based on THM has the advantages of high efficiency and stability and has broad application prospects in the fields of all-optical signal processing and high-speed optical communication.

## 1. Introduction

Since the theoretical feasibility of using optical fibers as a medium for information transmission was proposed, fiber optic communication has become an important component of modern communication systems. However, the modulation and detection of light signals transmitted through optical fibers are still limited by the rate of electro-optic conversion [1,2,3,4]. To meet the high-speed communication demands of contemporary society, all-optical modulators that integrate materials with different optical properties have been developed and have garnered attention due to their extensive applications in the fields of optical signal processing and beyond [5,6]. All-optical modulators can use pump light to directly control signal light, achieving modulation of the signal light’s intensity, phase, and polarization, among other characteristics [7]. Since the first demonstration of a graphene-based mode-locked fiber laser in 2009, a variety of physical mechanisms, including graphene cross-absorption modulation, rare-earth-doped fiber saturable absorbers, acousto-optic, and thermo-optic effects, have been applied to all-optical modulation [8,9,10,11,12,13,14]. Among them, the thermo-optic effect is one of the important mechanisms of optical devices, which causes the refractive index of the medium material to change with the variation in temperature. At the same time, due to the excellent light absorption performance and strong thermo-optic effect of thermo-optic materials, they are widely researched and used in the application of all-optical signal processing [15,16]. Thermo-optic modulation can be achieved through Mach–Zehnder interferometers or Michelson interferometers, etc. [17,18]. In 2015, researchers proposed an all-optical modulator assisted by the thermo-optic effect of graphene [19]. The heat generated by the interaction of graphene with the evanescent field of a microfiber causes an exponential change in the refractive index of the optical fiber, thereby causing a phase shift in the signal. Then, the all-optical switch with a modulation depth of 20 dB and a rise time of 9.1 ms has been realized [19]. By depositing a few layers of transition metal dichalcogenides and tungsten disulfide (WS_2_) onto a microfiber, an all-optical switch based on an optical fiber Mach–Zehnder interferometer phase shifter was achieved, showing a modulation depth of 15 dB and a rise time of 7.3 ms [20]. In 2018, some investigators utilized a microfiber with a few layers of phosphorene fluoride deposited on it to design a fully fiber-optic all-optical modulator. The modulator has a modulation depth of 17 dB and a rise time constant of 2.5 ms, and they successfully loaded the information carried by the pump light onto the signal light [21]. The common thermo-optic materials include transition metal dichalcogenides [22,23], graphene [24], and Au nanorods [25,26], etc. In order to further explore the potential of all-optical devices based on nanomaterials, researchers have conducted extensive studies on two-dimensional layered materials. They found that Ti_3_C_2_, as a two-dimensional transition metal carbide, has excellent thermo-optic properties and stability. The same as all MXene materials, Ti_3_C_2_ has a layered structure, and its optical absorption capacity and refractive index can be altered by adjusting the functional groups on its surface. This characteristic gives it very unique advantages in the fields of electrochemistry, optics, and so on [27]. Ti_3_C_2_, combined with other materials that have excellent optical properties, has become a new candidate material for the development of all-optical devices [28,29]. Heterojunction materials composed of layered Ti_3_C_2_ and TiN particles have been confirmed to exhibit high stability while also demonstrating high light absorption capacity and efficient thermo-optic effects [5,30]. Therefore, it is necessary to further research all-optical devices that incorporate TiN/Ti_3_C_2_ heterojunction materials.

In this work, the all-optical modulator that utilizes the thermo-optic effect of the TiN/Ti_3_C_2_ heterojunction has been reported. Couple the pump light with the signal light and propagate the coupled light into the THM composite structure. The TiN/Ti_3_C_2_ heterojunction absorbs the energy of the light, causing a change in the refractive index of THM, and finally induces a phase shift in the signal light. By adjusting the input power of the pump light through EDFA, a phase shift of the output spectrum of the signal light can be successfully observed, with a maximum phase shift of 4π and a phase shift slope of 0.025π/mW. Additionally, by using a narrowband optical source as the signal light and introducing pump light that has been intensity-modulated, thermo-optic modulation of the narrowband light can be achieved with a modulation depth of ~26.4 dB. Further, the modulation results can maintain relatively high stability and a deep modulation depth within a certain wavelength detuning range. It is expected that the thermo-optic modulator using TiN/Ti_3_C_2_ heterojunction-coated microfiber can show great potential in the fields of all-optical information processing fields.

## 2. Experimental Principle and Setup

In our previous work, we demonstrated the preparation methods of heterojunction material and carried out a series of characterizations on the heterojunction material [5]. Among the many 2D MXenes, Ti_3_C_2_ has attracted the attention of scholars due to its low cost, easy modification, and excellent electronic properties. TiN, as a transition metal material, has excellent optical properties in itself. Layered Ti_3_C_2_ MXene exhibits strong nonlinear saturable absorption characteristics in the near-infrared region, with a large nonlinear absorption coefficient and optical response. At the same time, the types of surface groups are adjustable, and its certain properties can be endowed or enhanced by changing the surface groups, which makes layered Ti_3_C_2_ have great potential in the field of optical communication. Therefore, it is of great significance to strengthen the research between layered Ti_3_C_2_ and other materials, such as TiN. At present, there is less research on all-optical devices composed of heterojunction materials of layered Ti_3_C_2_ with other materials with excellent optical properties. Therefore, the study of all-optical devices containing TiN/Ti_3_C_2_ heterojunction materials has high research value.

The complete preparation process of the THM composite structure is as follows: First, the TiN/Ti_3_C_2_ heterojunction was synthesized using an ultrasonic-assisted intercalation method. Then, the microfiber with a diameter of 6 μm was prepared using the process of flame-heated taper drawing [31]. This is because when the diameter is smaller, it is very prone to high-temperature melting. On the contrary, if the diameter is too large, the interaction between the laser and the material will be weakened. Finally, the THM composite structure was prepared by an optical deposition method [5]. The thermo-optic effect enables the THM composite structure to introduce a phase shift to the incident light signal, providing the possibility for the design of a thermo-optic modulator based on the Michelson interferometer.

**Figure 1:**
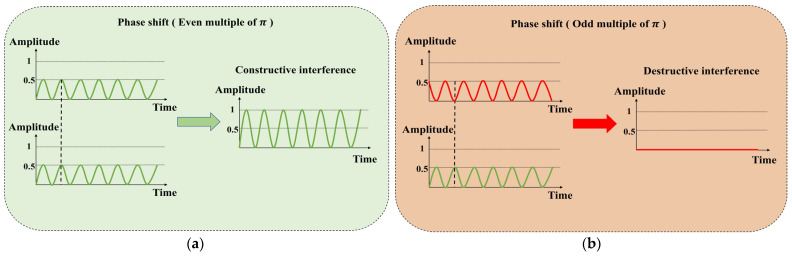
The principle of the Michelson-type all-optical modulator: (**a**) phase shift (even multiple of π); (**b**) phase shift (odd multiple of π).

Using the principle of the Michelson interferometer, the phase difference caused by the thermo-optic effect can be transformed into intensity modulation. Figure 1 illustrates the modulation principle based on the Michelson interferometer. The incident light signal is split into two paths, each with optical power equal to half of the original incident signal power. When these two paths of light are coupled and superimposed, they produce interference light. If the path parameters (such as optical path length) through which these two paths of light pass are different, this difference will create a phase difference between the two paths of light. When the phase difference is an even multiple of π, constructive interference occurs, resulting in the maximum output signal light amplitude. When the phase difference is an odd multiple of π, destructive interference occurs, leading to the minimum output signal light amplitude. Ultimately, based on the phase difference, interference light with varying optical intensities is output.

**Figure 2:**
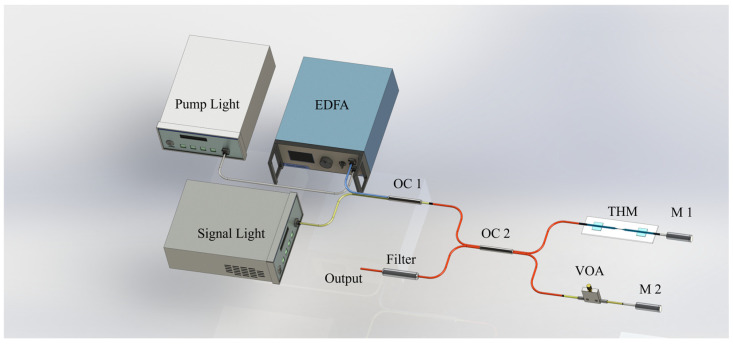
Schematic diagram of a Michelson all-optical modulator.

Based on the above principles, as shown in Figure 2, this paper designed the experimental setup of a Michelson-type all-optical modulator using THM. The device mainly consists of two parts: the light sources and a Michelson interferometer. The pump light source is a tunable external cavity laser (ECL), and an erbium-doped fiber amplifier (EDFA) is used to adjust the power of the pump light. The signal light source is an ASE light source and a tunable external cavity laser (ECL). These two light sources are used, respectively, to study the phase-shift performance and modulation performance of the device. The pump and signal lights are coupled into the interference device through optical coupler 1 (OC1). After passing through optical coupler 2 (OC2), the coupled beams are split into two paths with a 50:50 intensity ratio, entering the two arms of the Michelson interferometer, respectively. One path is equipped with a THM composite structure to control the phase of the signal light, and the other path has a variable optical attenuator (VOA) inserted to balance the optical power of the two beams. After being reflected by mirror1(M2) and mirror2(M2), the two beams interfere at OC2, then pass through a filter, and are finally output. The central wavelength of the filter at the output port is consistent with that of the signal light to filter out interference such as pump light. The output signal light is observed for phase shift using an optical spectrum analyzer (OSA). The time-domain waveform is observed using an optical oscilloscope.

**Figure 3:**
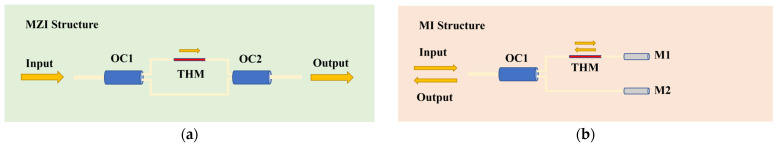
Schematic diagrams of MZI and MI structures. (**a**) MZI structure; (**b**) MI structure.

We choose the Michelson Interferometer (MI) over the Mach–Zehnder Interferometer (MZI) structure due to the differences in their experimental setups. As shown in Figure 3, when the optical signal enters the MZI structure, it is divided into two paths by the OC1 and then propagated through the two arms of the device. Up to this point, the propagation of the optical signal in both of the MZI and MI setups is the same. However, in the MZI, the two beams of light, after passing through the two arms of the device, are coupled and then output by OC2, whereas in the MI, the two beams of light are reflected by mirrors (M1 and M2) set behind the two arms and then return to the previous coupler to be output. This significantly extends the time that the optical signal acts within the two arms of the device, allowing for better utilization of the phase-shifting capabilities of the THM composite structure.

## 3. Results and Discussion

### 3.1. Phase Shift Experiment

**Figure 4:**
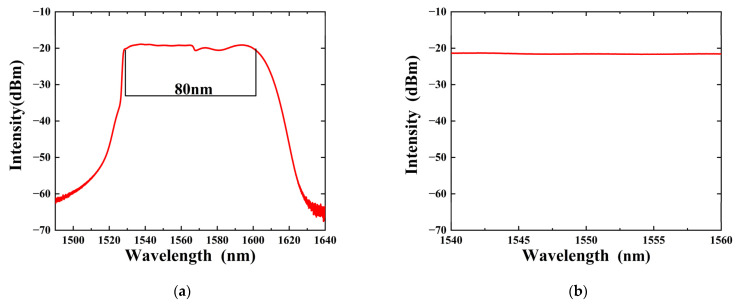
(**a**) Complete ASE light source spectrum; (**b**) C-band ASE spectrum.

To more intuitively observe the phase shift caused by the thermo-optic effect, an ASE light source is used as a signal source in this experiment. The ASE light source can produce a very broad spectral range. Within a specific wavelength range, the spectral intensity distribution is flat and stable. Figure 4a presents the complete ASE light source spectrum ranging from 1490 nm to 1640 nm. It can be seen that the ASE spectrum has a relatively flat intensity distribution over an approximate 80 nm wavelength range from 1525 nm to 1600 nm. The spectral lines within the range of 1540 nm to 1560 nm can be approximated as a straight line with a smooth intensity variation according to Figure 4b. This stable spectral characteristic can exclude the impact of intensity fluctuations of the ASE light source signal itself on the intensity variation in the output interference spectrum, which is beneficial for the observation and analysis of the interference spectrum in subsequent measurements.

The diameter of the microfiber is much smaller than that of the standard single-mode fiber, so when the laser is transmitted in the THM composite structure, the light wave is no longer completely confined within the fiber. Instead, a portion of the energy propagates along the axis direction on the surface of the microfiber in the form of an evanescent wave. This evanescent wave’s energy can be absorbed by the surrounding heterojunction material, thereby causing a change in the THM’s refractive index, which is known as the thermo-optic effect. After injecting the pump light, the TiN/Ti_3_C_2_ heterojunction material can rapidly absorb the energy of the pump light to produce local heat due to strong light-matter interaction, resulting in the phase shift of the signal light. Combined with the principle of interference, the phase shift caused by the thermo-optic effect of THM can be indirectly obtained through the movement of the spectrum.

**Figure 5:**
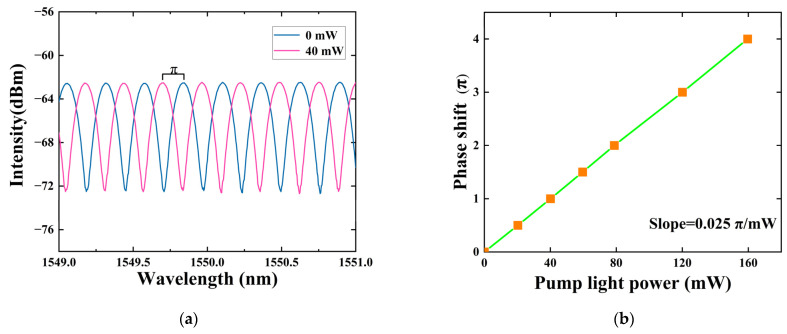
(**a**) Interference spectra of ASE light source at pump power of 0 and 40 mW; (**b**) the slope curve of signal light phase shift versus pump light power (after amplification by EDFA).

Through the EDFA, we can adjust the pump light to have different power levels. In the experiment, the phase modulation characteristics of the all-optical device based on THM were studied under different pump powers. In this experiment, the ASE light source power was set to 30 mW, and the pump light wavelength was fixed at 1553 nm. The blue line represents the interference spectrum when the pump light power is 0 mW in Figure 5a. It can be observed that after interference, the intensity of light at different wavelengths exhibits a sinusoidal pattern variation with wavelength. This is because the light of different wavelengths will experience different optical path differences in the interferometer, and thus exhibit different interference effects at the output end. Since the difference in this interference effect varies periodically with the change in wavelength, the ASE interference spectrum shows a sinusoidal pattern variation.

The red line represents the interference spectrum when the pump light power is 40 mW in Figure 5a. Compared to the interference spectrum at 0 mW, the waveform intensity in the output spectrum at this time changes from a peak (trough) to a trough (peak), which meets the condition in the interference principle where the intensity of the output optical signal changes from a maximum value (minimum value) to a minimum value (maximum value). At this point, the spectral phase shift can be estimated as π [15].

In the experiment, to obtain the relationship between the phase shift of the interference spectrum and the pump light power, the pump light power was further increased by the EDFA, and the intensity changes in the interference spectrum were observed. Figure 5b demonstrates the phase shift in the interference spectrum at various pump light powers. The phase shift of the interference spectrum exhibits a clear linear relationship with the pump light power. The calculated phase shift slope is 0.025π/mW. The conversion efficiency is superior to that of transition metal dichalcogenides (e.g., WS_2_, ~0.017π/mW) [20] and is close to that of phosphorene (0.029π/mW) [21]. The maximum phase shift of 4π can be achieved at the highest pump power (160 mW) in the experiment.

### 3.2. Modulation Experiment

**Figure 6:**
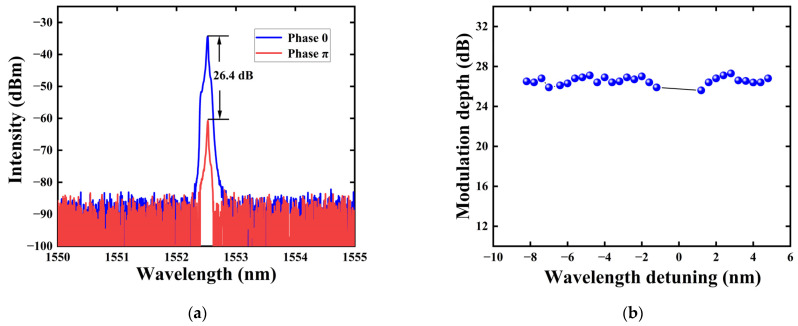
(**a**) Spectra of the signal light at phase shifts of 0 and π; (**b**) the relationship between the modulation depth of the signal light and wavelength detuning.

Then, the signal source can be replaced by the narrow-band light source (the ECL, which is mentioned in Section 2) in order to study the modulation depth of the all-optical modulator. The central wavelength of the filter at the output port is consistent with the wavelength of the signal light, which is used to filter out interference such as pump light. The pump light power is set to 0 and 40 mW, respectively, and the interference spectrum intensity output at phases of 0 and π is tested. Figure 6a shows the output interference spectrum intensity when the pump light power is 0 and 40 mW, respectively, from which it can be concluded that the modulation depth of the modulator at this time is 26.4 dB.

To study the impact of different wavelength detuning on the modulation depth, we fixed the wavelength of the incident signal light at 1552.09 nm and then changed the pump light wavelength. The effect of different wavelength detuning on modulation depth was tested with a wavelength interval of 0.4 nm. Figure 6b reflects the relationship between different wavelength detuning and modulation depth within a certain wavelength range. It can be observed that the modulation depth is almost maintained stably. We can see that the impact of wavelength detuning on the thermo-optic effect of THM is minimal. We concluded that even if the pump light wavelength and the signal light wavelength have a very small difference (>1 nm), the device still has a good modulation effect within this wavelength range. This indicates that the wavelength of the pump light has little impact on the modulation effect, while the laser intensity of the pump light has a significant impact on the modulation effect. Due to the limitation of the maximum wavelength tuning range of the ECL, the maximum range of wavelength detuning obtained in the experiment is approximately 16 nm, which is from −10 nm to +6 nm. At the same time, since the filtering range of the filter shown in Figure 2 is about 1 nm, sampling was not carried out in the wavelength detuning range of −1 to +1 nm in Figure 6b, in order to prevent errors caused by the signal light wavelength being close to the pump light wavelength.

The experimental results above indicated that due to the thermo-optic effect of the heterojunction material and the THM composite structure, when the pump and signal lights pass through the THM, a portion of the light’s energy will be absorbed by the heterojunction material, causing a change in the refractive index of the THM. This causes a phase change in the light passing through the THM, creating a phase difference with the light in the other path. When these two lights return to the OC2 due to reflection and then interfere, this phase difference can be converted into amplitude modulation. If the pump light introduced in the experiment is amplitude-modulated, then due to the thermo-optic effect and the principle of the MI, the signal intensity of the output light will show regular changes in response to the variations in the pump light intensity. Combined with the experimental results in Figure 5, by controlling the amplitude of the pump light intensity changes in the experiment, it is possible to vary the phase of the signal light passing through THM between 0 and π, thereby modulating the information carried on the pump light onto the signal light.

Specifically, we designed the thermal effect to produce a sufficient change in refractive index at specific bit symbols, thereby introducing enough phase difference to modulate the signal light. For example, when the bit symbol is “0”, the thermal effect causes a change in the refractive index of the THM, which leads to a “π” phase shift of the signal light passing through the THM path. At this point, the two light paths will interfere destructively at OC2, and the output amplitude of the interference light is at its minimum value. On the contrary, when the bit symbol is “1”, constructive interference will occur, and the output amplitude of the interference light is at its maximum value. Because of this, we can achieve amplitude modulation of the signal light using pump light with a non-return-to-zero (NRZ) bit sequence.

**Figure 7:**
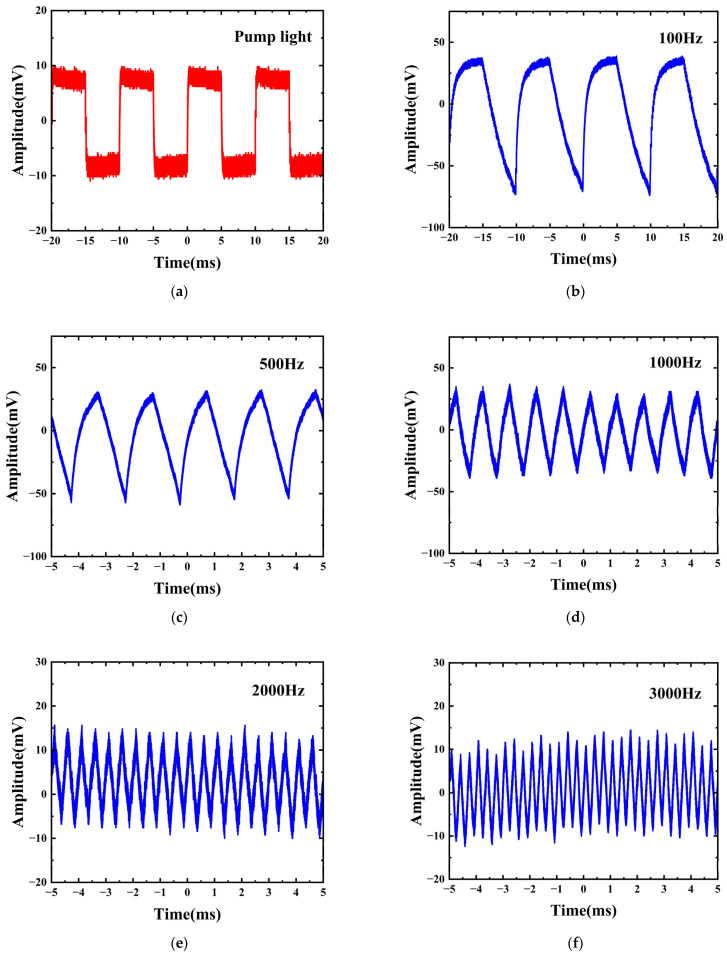
Waveforms of the signal light at different modulation frequencies. (**a**) Waveform of the pump light at 100 Hz. (**b**–**f**) Signal waveform at 100 Hz, 500 Hz, 1000 Hz, 2000 Hz, 3000 Hz.

An electro-optic modulator is used to modulate a narrow-band continuous light source, which serves as the pump light, into a 100 Hz NRZ rectangular wave as shown in Figure 7a. To achieve amplitude modulation, it is necessary to adjust the pump light power through EDFA. Before the EDFA operates, the power of two lasers was relatively small, so the impact on THM was minimal. After being amplified by the EDFA, the pump light power increases and reaches a level that satisfies the phase-shift condition. By calibrating the correspondence between the pump light amplitude and the phase shift of the output signal light, it can be arranged such that when the pump light carries high-level information (bit symbol “1”), the output optical signal is in a state of constructive interference, and when the pump light carries low-level information (bit symbol “0”), the output optical signal is in a state of destructive interference, thereby achieving amplitude modulation of the signal light. As shown in Figure 7b, the time-domain waveform of the output optical signal after modulation by the 100 Hz pump light can be observed, and the waveform width (10 ms) matches the pump modulation frequency (100 Hz), proving that the incident light has been successfully modulated into a pulse waveform that has the same bit sequence as the pump light. However, due to the slow response characteristic of the thermo-optic effect [20,21], the rising and falling edges of the measured signal waveform become smooth. To further investigate the dependence on frequency response, the modulation frequency is gradually increased from 500 Hz to 3000 Hz to observe the effects of different modulation rates on the waveform of the output signal and the modulation effect. Figure 7c–f, respectively, display the optical signal output waveforms of the modulator at modulation rates of 500 Hz, 1000 Hz, 2000 Hz, and 3000 Hz. It can be observed that as the modulation rate gradually increases, the amplitude of the output optical signal waveform gradually decreases, and the waveform also changes into a sharp triangular wave. The degree of signal distortion gradually increases, which may be due to the lag effect of heat generation and heat dissipation [20,21]. Within the repetition frequency range of 100 Hz to 3000 Hz, the peak voltage of the output signal also gradually decreases as the repetition frequency increases, indicating that the modulation depth is also gradually decreasing. In the experiment, the 3 dB modulation bandwidth of this modulator was measured to be approximately 2000 Hz. Table 1 summarizes the modulation effects of some existing all-optical modulators based on different materials. From this table, it can be seen that compared to other materials, the all-optical modulator using the TiN/Ti_3_C_2_ heterojunction has a deeper modulation depth and can meet the application scenarios that require higher modulation frequencies, proving its greater potential in the fields of all-optical signal processing and high-speed optical communication.

## 4. Conclusions

In summary, an all-optical modulator based on TiN/Ti_3_C_2_ heterojunction has been studied. The phase-shifting capability of THM under different pump powers was studied using an ASE light source. At a pump power of 160 mW, the interferometric spectrum achieved a maximum phase shift of 4π, demonstrating a significant thermo-optic effect. The phase shift shows a clear linear relationship with the pump light power, and the phase shift slope is 0.025π/mW. Compared to the state at 0 mW, when the phase shift reaches π, the modulation depth is 26.4 dB, which is deeper than that of graphene film, phosphorene and transition metal dichalcogenide structures. Moreover, the all-optical modulator based on the THM composite structure, using a fully fiber-optic Michelson interferometer, has a wider 3 dB modulation bandwidth (~2000 Hz). The results of this experiment are expected to enrich the research on all-optical modulators and may contribute to the design of more efficient all-optical signal processing optical communication systems.

## Figures and Tables

**Table 1:** Example of all-optical modulators in the reports.

Materials	Phase Shift Slope (π/mW)	Rise Time (ms)	Interference Principle	Modulation Depth (dB)	3 dB Modulation Bandwidth (Hz)	Refs.
Graphene	0.091	9.1	Mach–Zehnder	20	-	[19]
WS_2_	0.017	7.3	Mach–Zehnder	15	320	[20]
Phosphorene	0.029	2.5	Mach–Zehnder	17	270	[21]
MXene Ti_3_C_2_T_x_	0.061	4.1	Mach–Zehnder	>18.53	-	[32]
Bismuthene	0.053–0.076	3.42	Michelson	25	-	[33]
MXene Ti_3_C_2_T_x_	0.043	~5.3	Michelson	>27	-	[34]
Antimonene	0.049	3.2	Michelson	25	-	[35]
TiN/Ti_3_C_2_ heterojunction	0.025	-	Michelson	26.4	~2000	This work

## Data Availability

Data will be made available on request.
